# Investigating Structure and Dynamics of Proteins in Amorphous Phases Using Neutron Scattering

**DOI:** 10.1016/j.csbj.2016.12.004

**Published:** 2016-12-21

**Authors:** Maria Monica Castellanos, Arnold McAuley, Joseph E. Curtis

**Affiliations:** a NIST Center for Neutron Research, National Institute of Standards and Technology, 100 Bureau Drive, Mail Stop 6102, Gaithersburg, MD 20899, United States; b Institute for Bioscience and Biotechnology Research, 9600 Gudelsky Drive, Rockville, MD 20850, United States; c Department of Drug Product Development, Amgen Inc., One Amgen Center Drive, Thousand Oaks, CA 91320, United States

**Keywords:** Neutron scattering, Protein structure, Protein dynamics, Freeze-dried proteins, Glasses, Frozen protein solutions, Molecular dynamics

## Abstract

In order to increase shelf life and minimize aggregation during storage, many biotherapeutic drugs are formulated and stored as either frozen solutions or lyophilized powders. However, characterizing amorphous solids can be challenging with the commonly available set of biophysical measurements used for proteins in liquid solutions. Therefore, some questions remain regarding the structure of the active pharmaceutical ingredient during freezing and drying of the drug product and the molecular role of excipients. Neutron scattering is a powerful technique to study structure and dynamics of a variety of systems in both solid and liquid phases. Moreover, neutron scattering experiments can generally be correlated with theory and molecular simulations to analyze experimental data. In this article, we focus on the use of neutron techniques to address problems of biotechnological interest. We describe the use of small-angle neutron scattering to study the solution structure of biological molecules and the packing arrangement in amorphous phases, that is, frozen glasses and freeze-dried protein powders. In addition, we discuss the use of neutron spectroscopy to measure the dynamics of glassy systems at different time and length scales. Overall, we expect that the present article will guide and prompt the use of neutron scattering to provide unique insights on many of the outstanding questions in biotechnology.

## 1 Introduction

The structure and dynamics of biological systems in solid and liquid environments are of significant importance for applications in protein engineering and to investigate biological function and biochemical mechanisms. Structural changes could lead to protein misfolding and aggregation, which have been correlated with loss in functionality and pathogenic mechanisms [1], [2], [3]. Additionally, proteins are not static entities as they adopt different conformations at various time and length scales that can directly affect biological function and stability. Because the solvent plays a direct role in dynamic processes of protein solutions and dried proteins [4], [5], [6], it is of interest to explore the effect of the solvent on protein dynamics and its effects on protein function and long-term chemical and thermodynamic stability.

Neutron scattering can distinctively probe the structure and dynamics of almost all materials, including biological systems, as it is sensitive to the position and motions of atoms. The first neutron scattering experiments were performed in the 1940s, as part of the Manhattan Project, where the feasibility of using neutrons for determining the structure of materials was demonstrated [7]. Further advances in instrumentation have led to the development of neutron scattering as an important characterization tool in science. Clifford G. Shull and Bertram N. Brockhouse received the Nobel prize in 1994 for their “pioneering contributions to the development of neutron scattering techniques for studies of condensed matter” [8]. Currently, there are more than twenty neutron sources around the world [9], where thousands of researchers in various fields are using neutron scattering to get further insights into our understanding of the structure and dynamics of condensed matter.

Some of the properties of neutrons that make them useful for a wide range of applications in biotechnology include [10]:

1. Wavelengths ranging from 0.1  Å to 15  Å that allow the study of structures as small as atoms to biological cells.
2. A wide range of energy differences (nanoelectronvolts to electronvolts) can be probed when neutrons interact with matter; thus, neutrons are sensitive to processes such as folding and diffusion.
3. The scattering power varies randomly for different nuclei, and it can be significantly different for isotopes of the same element. This feature is clearly observed with hydrogen and deuterium; therefore, contrast techniques can be used to study different components of a multicomponent system.
4. Because neutrons do not have charge, they can easily penetrate ordinary matter; thus, diverse sample environments can be used without affecting the measurement. Samples are not perturbed or destroyed and can be recovered for additional analysis.
5. Neutron scattering data contain information on both the distribution of atoms and motions that are not readily accessible with most other characterization techniques.

**Fig. 1:**
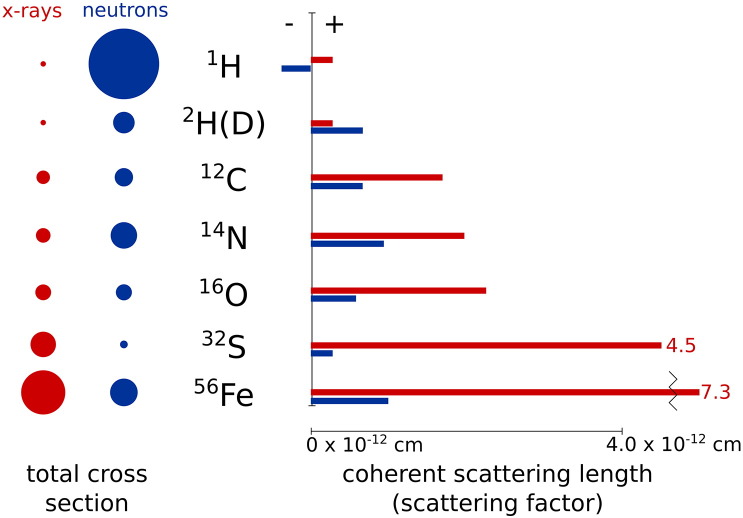
X-ray and neutron scattering cross sections and coherent scattering lengths (scattering factors) for different elements. Circles and bars are drawn to scale.

Source: X-ray cross sections at 100 keV are according to the values reported in reference [11] . Neutron cross sections are taken from reference [12] . Coherent scattering length and scattering factors are taken from reference [13].

One of the main advantages of neutrons is their selectivity to specific isotopes, which are not differentiated by photons (X-rays). This selectivity depends on the cross section of the atom *σ*, which represents the ratio of the outgoing current of scattered neutrons and the incident flux [14]. *σ* is related to the scattering length *b* as:

(1) $$\sigma =4\pi b^{2},$$

where *b* represents the apparent “size” of an element during a scattering event. For X-rays, *b* is often referred to as *f*(0). Biologically relevant atoms have distinct neutron cross sections and coherent scattering lengths as shown in Fig. 1, Table 1. Cross sections and coherent scattering lengths for X-rays are shown in Fig. 1.

As depicted in Table 1, each element has a coherent and incoherent neutron cross section, both of which determine the total scattering intensity of the system. The coherent contribution contains information on how the atoms are distributed in the sample; thus, the coherent scattering provides information about molecular shape and morphology. The incoherent cross section originates from the existence of the neutron spin. This incoherent contribution does not exist for atoms like ^12^C or ^16^O, but it is significant for hydrogen ^1^H. As depicted in Fig. 1, Table 1, hydrogen has a large neutron incoherent cross section compared to its isotope deuterium ^2^H(D) and other elements; thus, hydrogen contributes significantly to the incoherent and total scattering. Consequently, changing ^1^H by ^2^H(D) leads to a major effect on the measured scattering. On the other hand, hydrogen and deuterium scatter X-rays equally. Depending on the information that one wishes to extract, X-ray scattering can be useful as a complementary technique to neutron scattering. Moreover, because atoms are not rigidly bound, scattering events may induce a change in the energy of the neutron, which results in inelastic scattering events. This inelastic scattering contribution contains information on the motion of atoms, allowing the use of neutrons for dynamics measurements.

In this article, we focus on a set of biotechnological relevant problems that have been studied with the following neutron scattering techniques: small-angle neutron scattering (SANS) for structure, and quasielastic neutron scattering (QENS) for dynamics. These techniques are useful for gaining information on the structural arrangement and dynamics of proteins as well as establishing correlations with stability in amorphous phases. Readers interested in other neutron techniques, or other applications of neutron scattering in biology, are referred to general reviews discussing the fundamentals of neutron scattering [15], [16], [17], [18], [19], neutron scattering for structural biology studies [13], [14], [20], [21], and contrast matching techniques for biological samples [22], [23], [24], [25].

## 2 Overview of Small-Angle Neutron Scattering

**Fig. 2:**
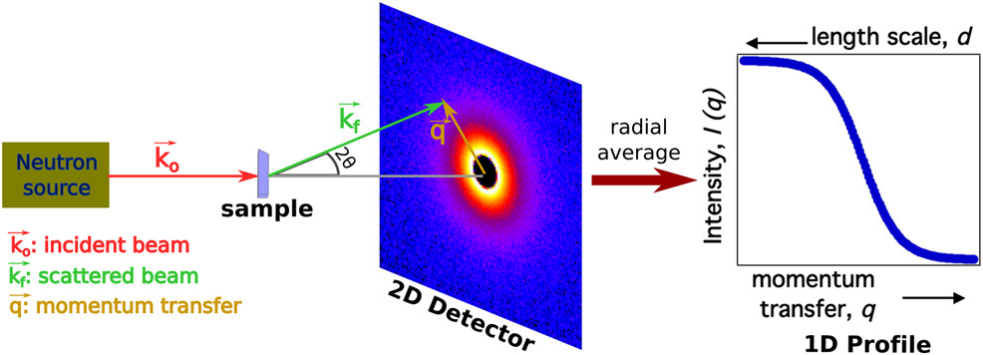
Schematic of a small-angle neutron scattering experiment.

Structural information from biological samples can be obtained using small-angle scattering, because the direction of the scattered neutrons depends on the relative position of atoms. A schematic of a SANS experiment and its notation are presented in Fig. 2. In SANS, neutrons with wavelength *λ* and wave vector ${\hat{k}}_{o}$, with magnitude $\frac{2\pi }{\lambda }$, interact with the sample, resulting in a scattered neutron wave vector ${\hat{k}}_{f}$ with scattering angle 2*θ*. As shown in Fig. 2, the momentum transfer $\hat{q}$ can be defined as $\hat{q}={\hat{k}}_{f}-{\hat{k}}_{o}$, and its magnitude $\left|\hat{q}\right|$, or simply *q*, corresponds to:

(2) $$q=\frac{4\pi \sin\theta }{\lambda },$$

which can be related to the probed length scale *d* as $q=\frac{2\pi }{d}$ using Bragg's law. Small-angle scattering is essentially governed by the same equations as Rayleigh light scattering, but the range of $\hat{q}$ can correspond to shorter length scales in SANS. Measurements at larger angles can be done to measure structural elements in the ‘wide-angle’ regime, which can provide valuable insight [26]. Such measurements can be limited by instrumentation details, incoherent background, and degree of order of structural elements at shorter length scales in solution.

The scattered intensity $I(\hat{q})$ is measured by a 2D detector. For samples with molecules randomly oriented, the 2D intensity can be radially averaged to obtain a 1D profile of the scattered intensity $I(\hat{q})$ as a function of *q*. $I(\hat{q})$ can be separated into a coherent $I_{coh}(\hat{q})$ and incoherent *I*_*incoh*_ contribution as follows:

(3) $$I(\hat{q})=I_{coh}(\hat{q})+I_{incoh}.$$

As shown in Eq. (3), the incoherent scattering is independent of $\hat{q}$. Although the incoherent scattering contribution, which comes mostly from hydrogen, can be estimated if the atomic composition of the sample is known, its effects are seen as a constant background or “flat region” at high *q*. At dilute concentrations, the incoherent scattering is mostly due to the buffer; thus, the coherent contribution from the sample can be obtained by subtracting the scattering of the buffer. In most cases, it is preferred that the solvent is deuterated to minimize the incoherent background. Moreover, the scattering of the buffer is generally independent of *q* in SANS. Therefore, for concentrated solutions, both the scattering of the buffer and the incoherent scattering can be approximated to a single constant value in the high *q* region of the scattering profile. Nevertheless, the scattering of the buffer must always be measured to confirm that it is *q* independent.

The coherent contribution to the scattering contains information on the spatial arrangement of atoms. For a pair of atoms *j* and *k* with scattering lengths *b*_*j*_ and *b*_*k*_, and positions ${\hat{r}}_{j}$ and ${\hat{r}}_{k}$ respectively, the coherent scattered intensity $I_{coh}(\hat{q})$ per unit volume *V* is expressed as:

(4) $$I_{coh}(\hat{q})=\frac{1}{V}\underset{j,k}{\sum }b_{j}b_{k}<\exp[i\hat{q}\cdot (\hat{r}j-{\hat{r}}_{k})]>,$$

where the sum is extended to all pairs of atoms in the system [14], [27], [28] .  <> represents an ensemble average, that is, the average position and orientation of atoms in the sample. In the following discussion, we only refer to the coherent contribution of the scattered intensity, or simply $I(\hat{q})$, which provides the structural information of interest.

For a collection of molecules with volume *V*_*p*_ at a volume fraction *ϕ*, $I(\hat{q})$ is written as:

(5) $$I(\hat{q})=\phi V_{p}\Delta \rho^{2}P(\hat{q})S^{\prime }(\hat{q}),$$

where $P(\hat{q})$ is the particle form factor and $S^{\prime }(\hat{q})$ is the effective structure factor [28]. *Δρ*, known as the contrast, depends on the scattering length densities $\rho =\frac{b_{mol}}{V}$ (average scattering length *b*_*mol*_ in a unit volume V) of both the molecule and the solvent as follows [14]:

(6) $$\Delta \rho =(\rho_{molecule}-\rho_{solvent}).$$

**Fig. 3:**
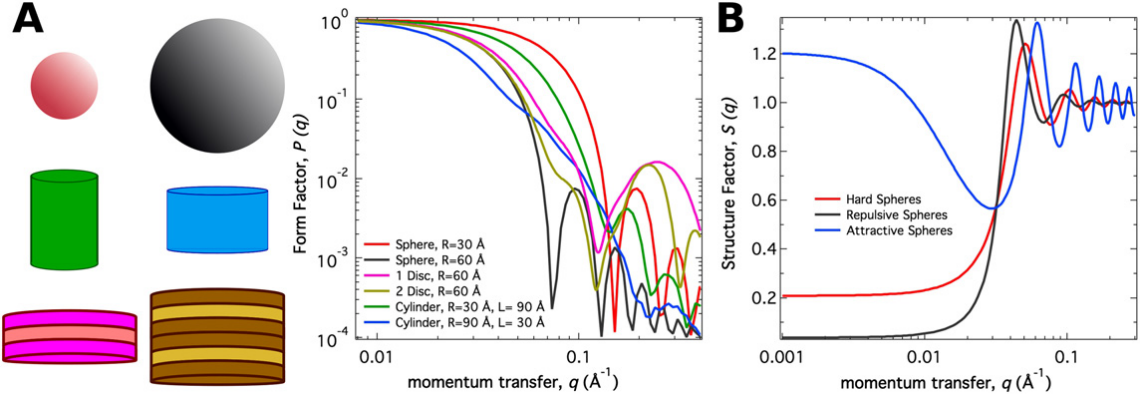
A. Form factor *P*(*q*) for various shapes. B. Structure factor *S*(*q*) for interacting spheres.

The significance of $P(\hat{q})$ and $S^{\prime }(\hat{q})$ is graphically presented in Fig. 3. $P(\hat{q})$ contains information on the position of the atoms within a single molecule; therefore, it is related to the shape and conformation of the molecule, that is, the intramolecular structure. By definition, $P(\hat{q})=1$ at *q* = 0.

On the contrary, $S^{\prime }(\hat{q})$ depends on the spatial correlations of atoms in different molecules, that is, intermolecular correlations. $S^{\prime }(\hat{q})$ is defined as an “effective” structure factor, because it is affected by the shape and anisotropy of interactions between molecules. For spherical particles with isotropic interactions, $S^{\prime }(\hat{q})$ is equivalent to the structure factor $S(\hat{q})$. By definition, $S(\hat{q})=1$ in the dilute case as molecules are not close enough to interact. However, as the particle concentration increases, $I(\hat{q})$ strongly depends on both intermolecular interactions and molecular shape. Under those conditions, $S^{\prime }(\hat{q})$ can be calculated by using Eq. (5) and the known $P(\hat{q})$ from the scattering of the dilute solution. Note that only $P(\hat{q})$ and $S^{\prime }(\hat{q})$ have a dependence on $\hat{q}$. Although this calculation assumes that the overall structure remains unaffected as concentration increases, differences in molecular conformation and flexibility do not affect the low $\hat{q}$ region of the scattering profile up to a $\hat{q}$ value that depends on the particular protein [29]. As depicted in Fig. 3B, changes in $S'(\hat{q})$ provide information on the net interparticle interactions that govern the system. Specifically, net repulsive systems have $S'(\hat{q})$ values less than one, whereas net attractive systems have $S^{\prime }(\hat{q})$ values larger than one [30], [31], [32], [33].

The remainder of this section briefly describes the advantages of SANS to study biological complexes and some of the computational tools available to analyze scattering data. This article is focused on the use of neutron scattering techniques in amorphous phases; therefore, the discussion is intended to be informative rather than an exhaustive review on the solution scattering analysis of biomolecules. Interested readers are referred to the reviews mentioned in the Introduction section.

Overall, SANS is largely used for structural biology studies, because it contains information on the conformation of biomolecules and protein–protein interactions. Under dilute conditions, the scattering profile is a signature of the molecular conformation in solution. If the coordinates of the atoms in the molecule are known, from either a crystallographic structure or a homology model, a scattering profile can be calculated to compare to experimental data. Moreover, molecular simulations can be used to obtain ensembles of structures, whereby scattering profiles can be calculated and used to model experimental SANS data. Tools, such as SASSIE [34], are available to generate ensembles of structures using molecular simulations and to compare simulated scattering profiles to experimental data. SasCalc (golden vector method) [35] allows the computation of neutron scattering profiles from structures and it can be freely accessed from a website [36]. Examples of this methodology are published in the literature [29], [34], [37], [38], [39], [40]. In the absence of crystallographic data, there are tools to generate molecular shapes that fit the experimental data, such as DAMMIF [41]. Nevertheless, small-angle scattering is a low resolution technique; thus, the outcomes of any model exclusively generated by experimental scattering data need to be interpreted with care and within the limitations of the technique.

**Fig. 4:**
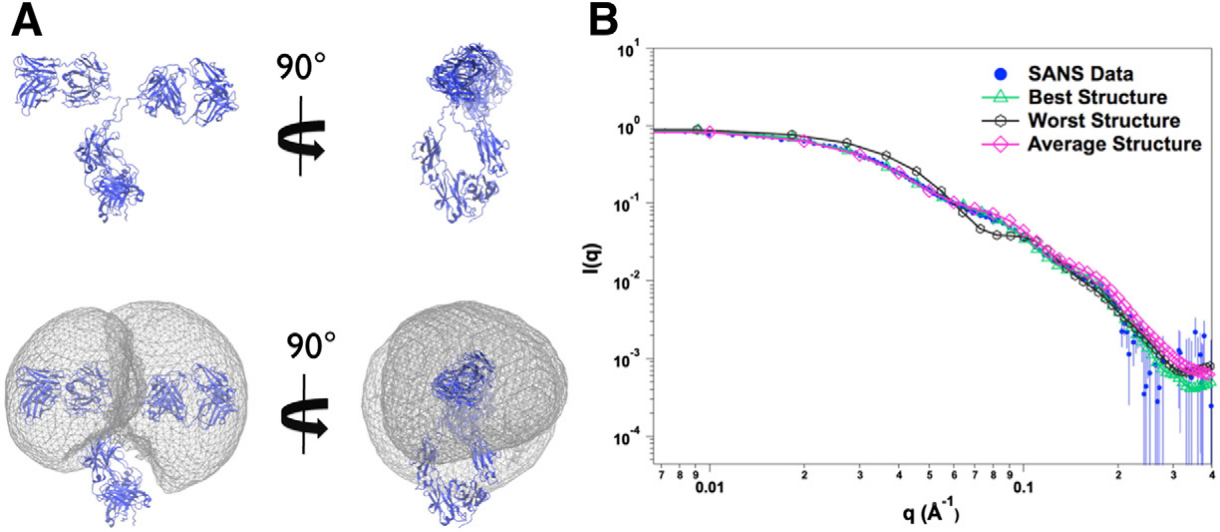
A. Different views of an atomistic monoclonal antibody structure and the corresponding ensembles represented by density plots. B. Scattering profiles are calculated for the ensemble of structures and compared with experimental data. Error bars represent ± 1 standard deviation.

Source: Adapted with permission from reference [37] . Copyright 2013 American Chemical Society.

This methodology of generating ensembles of structures and calculating scattering profiles has been applied to monomers [37] and dimers [42] of monoclonal antibodies (mAbs) in solution. mAbs and their derivatives are the fastest growing class of biotherapeutics and have been approved to treat various conditions, such as oncology, autoimmune and infectious diseases, among others [43], [44]. However, it is not entirely clear how the structure of these molecules affects their function, stability, and interactions. In contrast to the Fab and Fc, for which X-ray crystallography can be obtained, the hinge region is much more difficult to characterize. The hinge region mediates the flexibility of the molecule and the configuration of the Fab and Fc regions [45], [46]. Fig. 4 depicts an experimental study aided by molecular simulations on the solution structure of a mAb using SASSIE [34], [36]. To validate the simulated structures, scattering profiles are calculated and compared with experimental SANS data. Although one should be careful in assuming a single solution to the problem of modeling small-angle scattering data [37], SANS can provide a unique set of constraints not attainable with other techniques. These constraints can be used to reject structures or ensembles that do not describe experimental data.

Contrast variation studies have been used to study the structure of macromolecules in multicomponent systems. Examples of this methodology in biological systems are presented in the literature [25], [47], [48], [49], [50], [51]. The basic idea of this method is that the scattered intensity depends on *Δρ* (see Eq.(6)), that is, how well a molecule scatters with respect to the solvent.

**Fig. 5:**
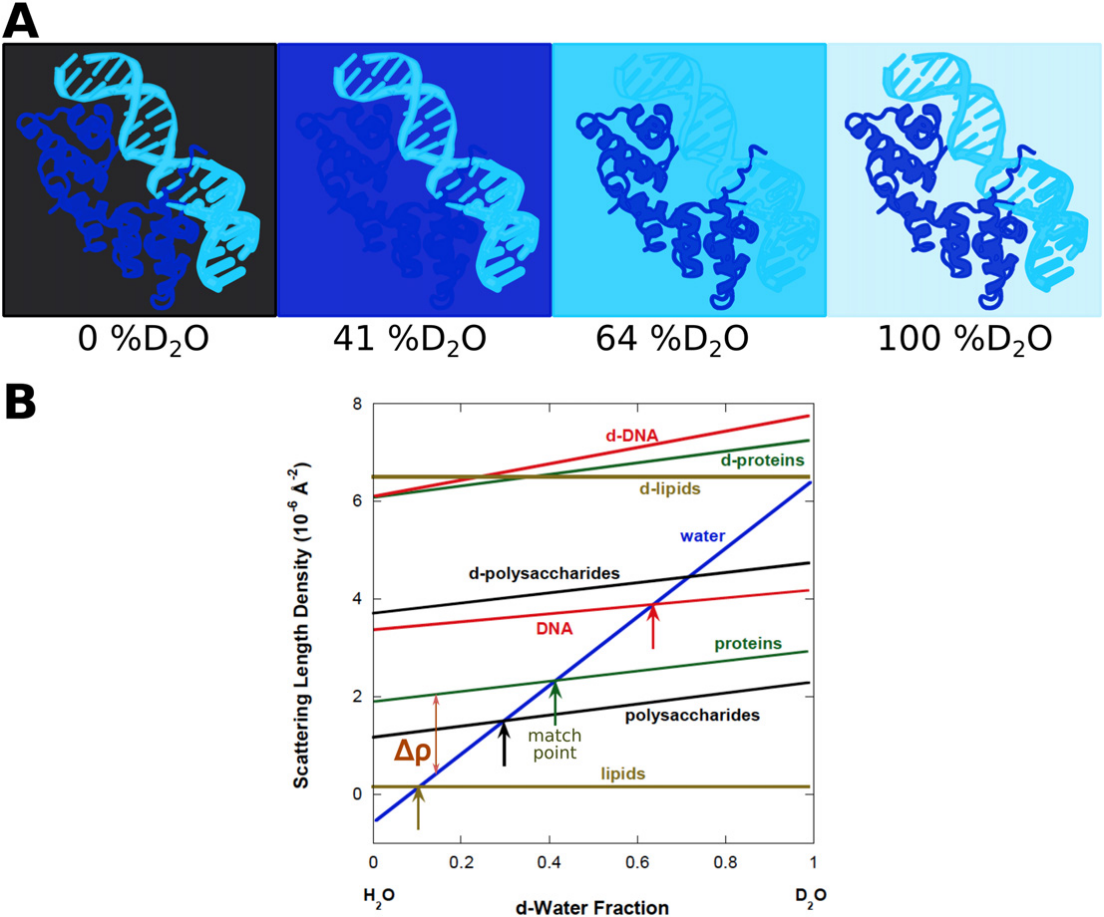
A. Schematic of a contrast variation experiment in SANS for a protein–DNA complex using different ratios of H_2_O/D_2_O. Protein–DNA structure was generated from PDB 1LMB [52] . B. Scattering length densities for various biomolecules were adapted from reference [28] with permission from the author.

The contrast variation technique is graphically represented in Fig. 5. As discussed previously, scattering length densities can significantly change by replacing atoms with their isotopes, particularly by changing ^1^H to ^2^H(D). As depicted in Fig. 5B, many labile non-aliphatic hydrogens in biomolecules can exchange in deuterated solvents, which changes *ρ*_*molecule*_. Note that isotopic labeling can affect the scattering length density and thus the contrast.

Fig. 5 shows that there is a percentage of *D*_2_*O* in the solvent for which *Δρ* = 0 that depends on the type of biomolecule. This is the contrast match point, where the molecule is not detected with neutrons. As an example, consider a complex consisting of a protein and DNA in an aqueous buffer. In a solvent with 0% *D*_2_*O*, both protein and DNA have a net contrast; therefore, both molecules can be distinguished from the solvent. If the *D*_2_*O* content is increased to ∼41% *D*_2_*O*, the contrast factor is zero for the protein and only DNA can be distinguished from the solvent. Similarly, if the *D*_2_*O* content is increased to ∼64%, neutrons do not distinguish between DNA and the solvent; thus, only the scattering from the protein component can be observed. By varying the *D*_2_*O* content, the individual components of a complex molecule can be studied. Alternatively, molecules can be selectively deuterated to change the scattering length density of the molecule. The contrast variation method is applicable for liquid and frozen samples.

Before describing some experimental results of SANS in amorphous phases, we briefly mention some practical considerations. For most protein solutions, a minimum concentration of 0.5  mg/mL is required for a SANS experiment. SANS is also used to study very concentrated samples up to the solubility limit of the components in the sample. Depending on the molecular weight of the protein and the sample concentration, a typical experiment takes about 20–60 min per sample. The scattering data can be reduced using SANS reduction macros developed at the NIST Center for Neutron Research [53], or other routines available at other neutron scattering facilities, such as Oak Ridge National Laboratory (Tennessee, USA) and the Institut Laue-Langevin (Grenoble, France). The importance of sample preparation and characterization for a small-angle scattering experiments has been discussed in the literature [54], [55].

## 3 SANS in Amorphous Phases

Many therapeutic proteins and pharmaceutical products are formulated as freeze-dried powders or frozen solutions to improve their long-term stability [56], [57]. In addition, freezing is the first step in the lyophilization process of a protein and it impacts both process performance and product quality [57]. Therefore, understanding how proteins are arranged in amorphous phases at the molecular level and correlating the morphology and dynamics of packed proteins with their stability profiles can provide new insights to better design and formulate frozen and lyophilized products. Additionally, proteins may adsorb on interfaces formed during freezing and drying, which could lead to partial unfolding and changes in secondary structure [58]. Such processes may be responsible for formation of high-molecular-weight aggregates and particles [59], [60].

Although numerous biophysical characterization techniques require samples to be in solution, neutron scattering is a versatile technique that allows the study of proteins in either liquid or solid phases. In particular, both lyophilized powders and frozen glasses of the protein lysozyme have been studied with SANS [61], [62], [63], [64]. Moreover, SANS can provide information on the morphology and heterogeneities in glassy matrices on nanometer and submicron length scales [63].

If the equipment is available at the facility where neutron measurements are performed, cooling and warming processes can be performed in situ during the neutron measurements. Some of the questions that SANS can address include:

1. How close together do proteins pack upon freezing?
2. What structural changes occur as the temperature drops below the freezing point?
3. How does the initial protein concentration of a liquid solution affect the freezing process?
4. Is the structure of a protein maintained during freezing/thawing cycles?
5. How do excipients affect packing and structure?

**Fig. 6:**
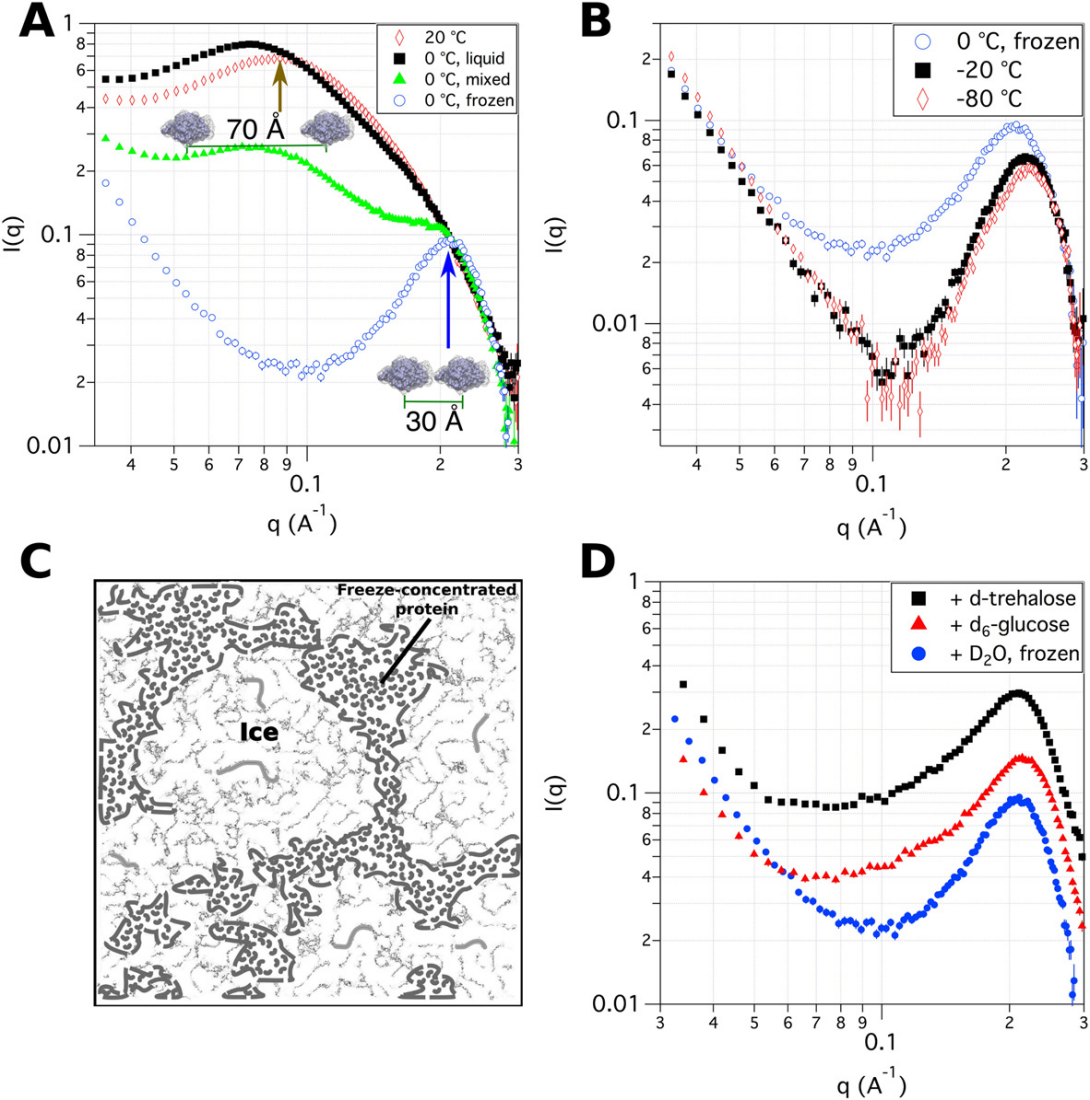
SANS profiles of lysozyme in amorphous phases. A. SANS profiles of lysozyme solutions during the freezing process. B. SANS profiles of frozen lysozyme solutions as a function of temperature. C. Schematic representation of the morphology of lysozyme in frozen solutions. D. SANS profiles of lyophilized lysozyme powders with controlled water content. Error bars represent ± 1 standard deviation.

Source: Adapted with permission from reference [61] . Copyright 2012 American Chemical Society.

Fig. 6A depicts the SANS profiles of lysozyme upon freezing a liquid solution. A peak in the scattering profile represents an increase in the number of correlations at a particular *q*_*peak*_, where the average separation distance between molecules is *d*_*peak*_ = 2*π*/*q*_*peak*_. At *q* ∼ 0.09  Å^ −1^, the peak is observed for a 100  mg/mL solution, which represents an intermolecular separation distance of 70  Å. As the temperature is decreased from room temperature to 0 ° C, the solution peak vanishes while a new peak emerges at a higher *q* ∼ 0.2  Å^ −1^, that is, a distance of ∼30  Å. Note that the radius of gyration of lysozyme is 14.3  Å [65]. As the temperature is decreased below the freezing point, the peak becomes sharper and slightly shifts to higher *q* values, which indicates that most proteins are packed closer together with decreasing temperature. This result is independent of the initial protein concentration in the starting solution and contrary to the behavior observed in solution [61], [62]. Consequently, most proteins are excluded from the water-rich phase (ice), forming a freeze-concentrated protein phase where the protein concentration can be in the order of ∼600  mg/mL for lysozyme. This exclusion of the protein from the ice phase is consistent with Raman and NMR experiments [66], [67]. The packing distance between proteins may affect the long-term stability of the drug substance and reconstitution times of lyophilized products.

The packing distance between proteins was reported to be independent not only of the protein concentration in the starting solution but also of the cooling rates studied [61], [62]. However, quickly cooling the protein solution from 20 ° C to −80 ° C leads to non-uniformities in the 2D scattering profile (not shown here). The non-isotropic nature of the 2D profile suggests a deformation of the sample on length scales larger than those probed by SANS.

Moreover, the overall structure of lysozyme remains unaffected after performing three freezing/thawing cycles, as the SANS profile in solution was fully recovered. Nonetheless, this is not the case for lysozyme in the presence of 0.4  mol/L NaCl, most likely due to polydispersity and phase separation [62]. These examples show the capabilities of SANS to evaluate the effect of freezing on the protein structure and how this process can be affected by excipients.

An additional feature of the scattering profile is an increase in the scattering at low-*q* in frozen samples and freeze-dried powders, which is absent in the measurements of lysozyme in solution. Using contrast variation experiments [61], [62], it was found that the scattering at low-*q* is a result of ice-cracks and proteinaceous aggregates that form during the freezing process. These ice-cracks consist of new solid/air interfaces that could induce structural changes in the secondary or tertiary structure of proteins. It should be noted that lysozyme aggregates formed during the freezing process were not present after thawing.

Although there are currently no capabilities to study the lyophilization process in situ, SANS can be used to study the product after lyophilization. Typically ∼0.4 mL volume (∼200  mg) of freeze-dried material is required for a SANS experiment. Fig. 6D shows the profile of lyophilized powders. A comparison with SANS profiles of frozen samples shows that the peak position remains invariant after the drying process. However, adding cryoprotectant excipients (saccharides) affects the packing distance of the lyophilized powder. The similarity of the SANS profiles after freezing and lyophilization suggests that the freezing process is the determining step on the protein–protein interactions of freeze-dried powders. Note that deuterated sugars may be necessary for the SANS measurements, because these excipients have a high hydrogen content that contributes to a significant incoherent background.

**Fig. 7:**
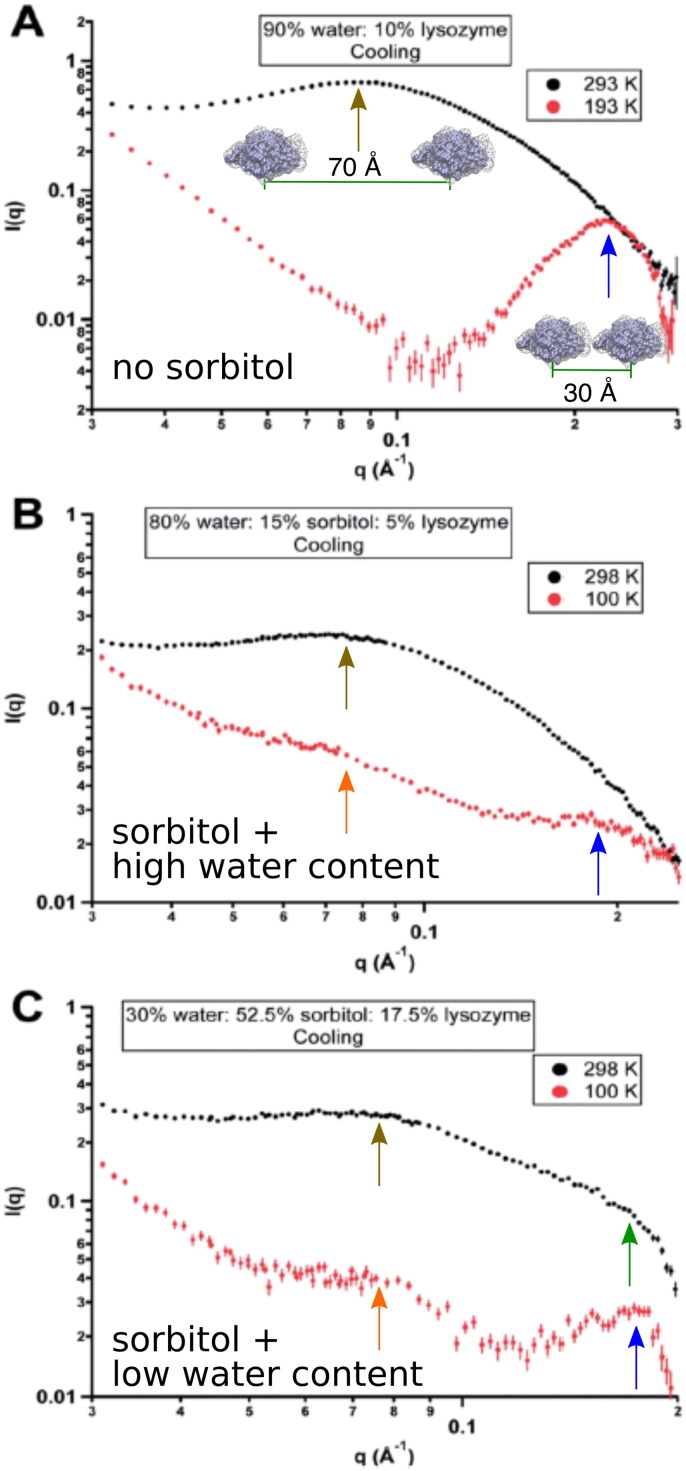
SANS profile of sorbitol–lysozyme frozen solutions. A. No sorbitol. B. Sorbitol & high water content. C. Sorbitol & low water content. Water refers to D_2_O. Protein to sorbitol ratio is equivalent in samples containing sorbitol. Error bars represent ± 1 standard deviation.

Source: Figure is adapted from reference [64] . Copyright 2014 Royal Society of Chemistry.

SANS can also be used to assess the effect of excipients, in particular cryoprotectants, on the packing morphology of frozen solutions [64]. Fig. 7 displays the effect of sorbitol on the packing morphology of frozen lysozyme solutions. SANS experiments show that the addition of sorbitol to lysozyme solutions decreases protein crowding in the frozen state, not only because the high *q*_*peak*_ becomes broader and slightly shifts to lower *q* (larger distances), but also because an intermediate peak emerges at *q* ∼ 0.07  Å^ −1^. Note that the position of this intermediate peak is independent of the initial protein concentration in solution, suggesting that clusters with uniform spacing form in the ice-phase. Therefore, phase separation is still observed, but the protein is excluded less from the ice-phase than in the case with no excipients (Fig. 6). The shift of the peak position to lower *q* in frozen lysozyme solutions with sorbitol indicates that proteins are further apart, and they are less likely to form high-molecular-weight species than in the absence of sorbitol.

## 4 Overview of Neutron Spectroscopy

Up to this point, we have focused our discussion on the structure and ordering of biological systems in amorphous phases. However, neutron scattering can also be used to study the dynamical properties of different components in the system. Changes in the motion of atoms are detected by a change in the velocity, and thus the energy, of the neutrons scattered by the sample. These techniques that measure the energy of the neutrons are known as neutron spectroscopy, which include inelastic neutron scattering and quasielastic neutron scattering (QENS). Inelastic neutron scattering corresponds to finite energy transfers. QENS is a limiting case of inelastic scattering where the energy transfer is small compared to the energy of the incident neutrons. In QENS, the energy transfer correspond to interactions of neutrons with particles diffusing in the femtosecond to nanosecond time scale[68]. The neutron spectra are generated by measuring the energy distribution spectrum of the scattered neutrons.

For a typical neutron spectroscopy experiment, hundreds of milligrams of sample are required and data acquisition for a single sample can take more than one day to measure. Data reduction software is available to process raw neutron spectroscopy data [69] and variants are used at all neutron scattering facilities.

The advent of neutron scattering methods has provided the largest amount of experimental data concerning protein dynamics below and through the glass transition temperature. One of the advantages of neutron scattering experiments is that they probe atomic motions on the 0.1 to 10^4^  ps time scale that allows for direct comparison to molecular dynamics (MD) simulations. Additionally, neutron scattering simultaneously measures dynamical events as a function of momentum transfer that correspond to length scales obtainable by MD simulations. Analysis of scattering experiments with MD simulations can be used to gain insight on the microscopic origins to the underlying dynamical behavior. Neutron spectroscopy experiments and MD simulations are used together in the remainder of this article; thus, a brief description of the underlying theoretical aspects is discussed below.

Neutron scattering offers a unique ability to probe structural and dynamical aspects of matter. The essential element of current neutron scattering experiments, in the context of studying protein dynamics, is the use of so-called cold neutrons with energies on the order of condensed phase excitation energies (∼25  meV) and wavelengths comparable to molecular dimensions (*λ* ∼1→ 20  Å) [28]. Although the wavelength of X-ray scattering is comparable to the neutron wavelength, the energies are typically much higher in X-ray experiments ( >2 keV). In a typical neutron spectroscopy experiment, one measures both energy (*Δ*E = E_*o*_ − E_*f*_ = ℏω) and momentum transfer ($\hbar \hat{q}=\hbar [{\hat{k}}_{f}-{\hat{k}}_{o}]$) of the neutron beam after passing through a sample. ℏ corresponds to the reduced Planck constant and *ω* is the frequency of the neutrons.

The scattering of a flux of neutrons is dependent upon the total scattering cross section of the sample, as discussed in previous sections. Changes in energy and momentum transfer are observed through the double differential cross section:

(7) $$\frac{\partial^{2}\sigma }{\partial \hat{\Omega }\partial E}=\frac{1}{\hbar }\frac{\partial^{2}\sigma }{\partial \hat{\Omega }\partial \omega },$$

which is the probability that a neutron passes through the solid angle $\hat{\Omega }$ with an energy E${}_{i}\pm \hbar \omega$. The double differential cross section is proportional to the sum of contributions from the coherent and incoherent dynamic structure factors:

(8) $$\frac{1}{\hbar }\frac{\partial^{2}\sigma }{\partial \hat{\Omega }\partial \omega }=\frac{{\hat{k}}_{f}}{{\hat{k}}_{o}}\left[\sigma_{coh}S_{coh}(\hat{q},\omega )+\sigma_{inc}S_{inc}(\hat{q},\omega )\right].$$

Coherent motion represents both diffraction and collective motions of all atoms in the sample, whereas incoherent motion is related to the single-particle dynamics (vibrations and diffusive motion) of individual atoms. The sample composition dictates the nature of the resulting scattering, whether it is coherent, incoherent or both. The resulting scattering can be directly linked to the respective cross sections *σ*_*inc*_ and *σ*_*coh*_, as previously shown in Table 1. For neutron spectroscopy, the most inherently useful aspect of the relative scattering cross sections is the unusually large incoherent cross section of ^1^H. Thus, neutron scattering experiments have the ability to discriminate single ^1^H motions in proteins thereby filling an experimental gap not accessible by X-ray experiments.

There are three main types of incoherent scattering events to consider. Elastic scattering E_*f*_ = E_*o*_, quasielastic scattering (E_*f*_ − E_*o*_)< 2  meV centered around the elastic line, and inelastic scattering (E_*f*_−E_*o*_) > 2  meV. Elastic scattering probes localized motions, quasielastic scattering probes diffusive motions, and inelastic scattering probes vibrations.

The natural variables of the neutron scattering experiment, $\hat{q}$ and *ω* are contained in the dynamic structure factor $S(\hat{q},\omega )$:

(9) $$S(\hat{q},\omega )=\frac{1}{2\pi }\int {\;}_{\tau }I(\hat{q},t)e^{-i\omega t}dt.$$

However, the connection to molecular dynamics simulations is through the incoherent intermediate scattering function $I(\hat{q},t)$:

(10) $$I(\hat{q},t)=\frac{1}{N}\underset{j}{\sum }\langle e^{i\hat{q}{\hat{r}}_{j}(t)}e^{-i\hat{q}{\hat{r}}_{j}(0)}\rangle ,$$

which is sampled from the MD trajectory $\left\{{\hat{r}}_{j}(0),\ldots ,{\hat{r}}_{j}(t)\right\}$ for all relevant particles *j*. Thus, the Fourier transform of $I(\hat{q},t)$, appropriately sampled over several values of $\hat{q}$ and trajectory points $\left\{{\hat{r}}_{j}(0),\ldots ,{\hat{r}}_{j}(t)\right\}$, can be performed to generate synthetic neutron scattering spectra $S{\left(\hat{q},\omega \right)}_{synthetic}$ after convoluting with the appropriate instrumental resolution function *R*(*ω*) to compensate for the finite experimental energy resolution such that:

(11) $$S{\left(\hat{q},\omega \right)}_{synthetic}=S(\hat{q},\omega )⊗R(\omega ).$$

**Fig. 8:**
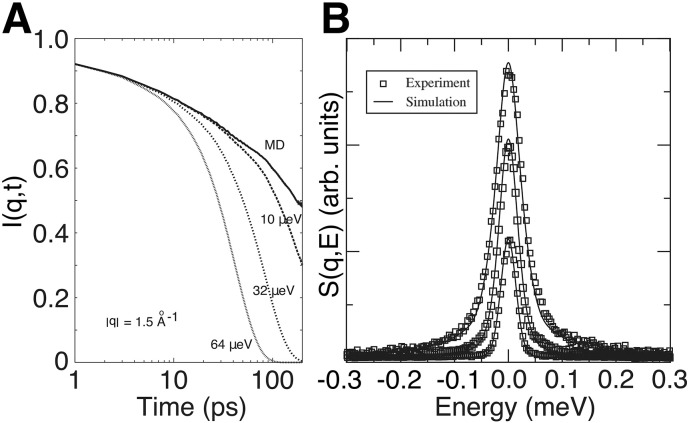
A. Theoretical intermediate scattering function $I(\hat{q},t)$ of non-exchangeable protons calculated directly from a molecular dynamics trajectory of the protein myoglobin in solution and after multiplying the MD result by various resolution functions corresponding to different instrumental resolutions. B. Incoherent dynamic structure factors $S(\hat{q},E)$ of the protein *α*-lactalbumin at selected magnitudes of momentum transfer ($\left|\hat{q}\right|=$0.5, 1.0, and 1.5 Å^ −1^ from bottom to top) measured by quasielastic neutron scattering (boxes) and predicted from MD simulations of the protein in solution (lines).

Source: Reprinted from reference [72] with permission from Elsevier.

Accounting for experimental resolution from MD simulations is achieved by $I(\hat{q},t)\cdot R(t)$, where *R*(*t*) is the experimental resolution function and applying the Debye–Waller factor [70], [71]. Examples of such corrections applied to $I(\hat{q},t)$ are shown in Fig. 8A. Although $S{\left(\hat{q},\omega \right)}_{synthetic}$ can be informative, the fine structure of the intermediate scattering function $I(\hat{q},t)$ can allow for a detailed comparison of experimental and theoretical results. An example of $I(\hat{q},t)$, $I(\hat{q},t)\cdot R(t)$ and $S{\left(\hat{q},\omega \right)}_{synthetic}$ and comparison to results from MD simulations and experiments are shown in Fig. 8.

The mean square displacement  <*x*^2^ >, also referred to as  <*u*^2^ > in some publications, is an integrated quantity that can be calculated from MD trajectories in at least two ways. The most direct method often reported is to simply average the atomic displacements over N atoms and *τ* time steps:

(12) $$<x^{2}>=\frac{1}{N\tau }\overset{N}{\underset{i=1}{\sum }}\overset{\tau }{\underset{t=1}{\sum }}(x_{i}(t)-x_{i}(0))(x_{i}(t)-x_{i}(0))$$

often utilizing multiple time origins [73]. To obtain quantitative agreement with neutron scattering spectra, one can calculate  <*x*^2^ > by obtaining $S(\hat{q},0)$ using Eqs. (9) and (10) and plotting $S{\left(\hat{q},0\right)}_{synthetic}$ versus ${\left|\hat{q}\right|}^{2}$ for a representative set of $\hat{q}$, whereby the resulting slope, $-\left|\hat{q}\right|<x^{2}>$, is used to obtain  <*x*^2^ >. This is valid under the assumption that the single particle displacements obey an isotropic Gaussian distribution as ${\left|\hat{q}\right|}^{2}\to 0$. This method naturally takes into account the finite energy resolution of the neutron scattering measurement. At a given $\hat{q}$, the corresponding length $∼\frac{2\pi }{q}$ can be considered to be the probability that a given particle is within the length scale of the measurement or calculation within the time/energy resolution of the instrument. Further informative descriptions of the calculation of  <*x*^2^ > from MD trajectories and the relationship between the analysis of neutron scattering data and MD simulations can be found in the literature [74], [75], [76].

## 5 Neutron Spectroscopy to Study Dynamics in Amorphous Phases

The addition of extraneous molecular additives, either as a cosolvent or as a cosolute, can have a dramatic impact on the dynamics and function of biological molecules. Cryoprotectants are commonly used in the biotechnology industry to prolong the achievable shelf-life of a compound and decrease the number of undesirable degradation products [77]. Common cryoprotectants are disaccharides, such as sucrose and trehalose, and polyols, such as glycerol. Although much is known about the ability of these excipients to alter the stability of biological molecules, a consistent molecular understanding of their effects on protein dynamics and stability has begun to emerge [78], [79], [80], [81]. A complete theoretical understanding emerging from molecular dynamics simulations is lacking at present, but it is an active area of research [82], [83], [84], [85].

The simulation of proteins in non-aqueous solvents is a challenging and exciting prospect. Although the computational chemistry community has made tremendous progress in the parameterization of force-fields for the simulation of proteins in homogenous aqueous phases, heterogeneous biomolecular systems require further force-field development [86]. Significant progress has been made in the parameterization of carbohydrates [87], [88] and MD studies of heterogeneous protein–carbohydrate systems have been published [89], [90].

For the remainder of this section, we focus upon a few seminal studies with broad impact upon the biotechnological industry that involve the study of the elements to suppress protein dynamics with a direct connection to the long-term stability of protein drugs. Therefore, this article is not meant to be an inclusive review of the extensive literature of the measurement of protein dynamics using neutron scattering. Interested readers are encouraged to consult classical papers [91], [92], [93], [94], reviews [76], [95], [96] and exciting neutron scattering studies involving hydration [71], [97], [98], [99], [100], [101], [102], [103], [104], [105], and important studies of dissacharides and polyols in solution [106], [107] and with proteins [83], [108], [109].

In particular, we highlight several studies that explore the role of glycerol and trehalose on protein dynamics and stabilization using neutron scattering and molecular dynamics simulations. An interesting pair of studies investigating the dynamics of protein powders in the presence and absence of glycerol using neutron scattering were reported by Tsai et al. [110], [111] . In the first of these studies, Tsai et al. [110] studied both the stabilization and destabilization of lysozyme powder as a function of hydration and glycerol content under different temperature regimes. The first regime studied was below and above a dynamical transition temperature T_*d*_ [91], which can be determined from a change in the slope of  <*x*^2^ > with temperature. Above T_*d*_, anharmonic motions are observed as a result from structural relaxations within the system. The second regime studied was below and above the thermal melting temperature of the protein T_*m*_, also known as the heat denaturation temperature. Hydrated lysozyme exhibits a dynamical transition near 200  K, which is typical of many other proteins. A T_*d*_ is *not* observed in dehydrated lysozyme powder from 50 K to 450  K; thus, this study provides further evidence of the importance of water to catalyze the protein structural relaxation. Moreover, the addition of increasing amounts of glycerol (20% and 50%) causes a decrease in T_*d*_ and an increase in the amplitude of anharmonic motions relative to the hydrated protein. Careful analysis, both below and above T_*d*_, indicates that increasing amounts of glycerol results in a stabilization effect below T_*d*_ and a destabilization effect above T_*d*_. This conclusion was reached by noting the lower mean square displacement amplitudes below T_*d*_ and the larger anharmonic mean square displacement amplitudes above T_*d*_.

In terms of thermal stability, Tsai et al. [110] observed that dehydrated lysozyme is more stable (T_*m*_ = 429  K) than hydrated lysozyme (T_*m*_ = 343  K). The authors also studied the effect of glycerol and they found that the addition of glycerol to dehydrated lysozyme has a destabilizing effect by lowering the T_*m*_, whereas the addition of glycerol to hydrated lysozyme has a stabilizing effect by raising the T_*m*_. In the second study [111], the authors found that the addition of glycerol to a partially hydrated protein powder (lysozyme or RNase A) leads to restricted motion, which is determined from an analysis of QENS using a stretched exponential function, compared to the hydrated protein powder alone.

Caliskan et al. [112], [113] used low-frequency Raman spectroscopy to study the dynamics of lysozyme in glycerol and trehalose. Interestingly, the authors found that, at low temperatures, liquid glycerol suppresses the fast conformational motions of the protein more than glassy trehalose. These results suggest that solvent viscosity is not the only important factor in the suppression of dynamical motions in proteins; i.e. specific solvent–protein interactions are also important. Sokolov and Gregory summarized this idea in their work on proteins and DNA in a review article [114].

**Fig. 9:**
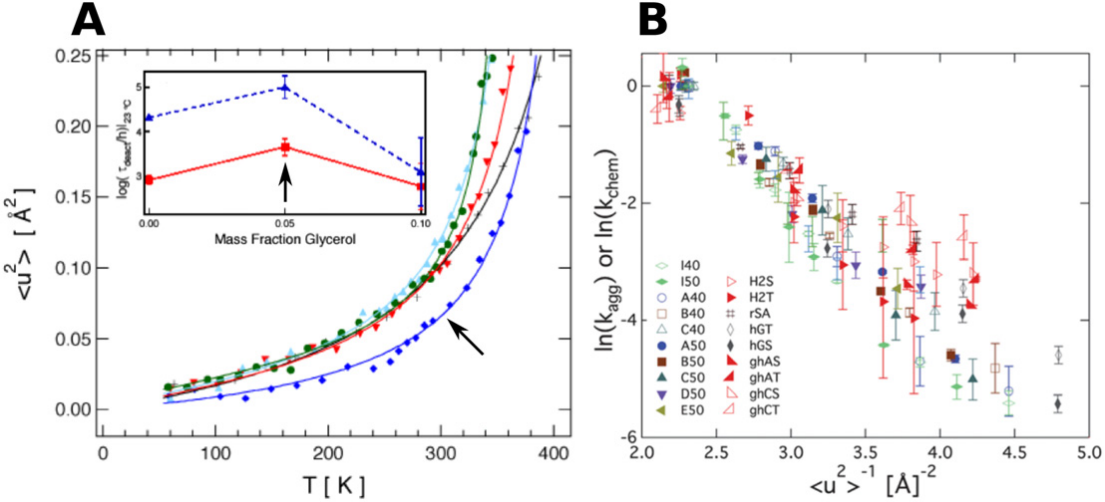
A.  <*u*^2^ > from neutron scattering of binary trehalose glasses as a function of glycerol mass fraction. Inset shows enzyme deactivation times in trehalose glasses with varying glycerol content. Higher deactivation times are observed at the same glycerol fraction at which  <*x*^2^ > is smallest at most temperatures, as pointed by the black arrows. Error bars represent ± 1 standard deviation. B. Correlation between  <*u*^2^ > and aggregation and chemical degradation rates of freeze-dried proteins in sugar glasses is from Ref. [81] . Each label represents a different protein and/or sugar matrix for either aggregation or chemical destabilization.

Source: Figure is adapted from references [81], [115] . Used with permission from Elsevier and the Royal Society of Chemistry.

Cicerone and Soles [115] reported elastic and inelastic neutron scattering of binary glasses of trehalose and glycerol. They found that an addition of a small mole fraction of glycerol (low T_*g*_) into trehalose (high T_*g*_) greatly suppresses the short length scale picosecond dynamics of the binary glass (Fig. 9A). Additionally, Cicerone et al. [116] measured the biological activity of several dilute enzyme preparations after storage in analogous binary glass systems. The authors found a strong correlation between restricted fast dynamics ( <ps) in the host glass with recoverable biological activity from analogous systems. This result suggests that screening cosolvents for their picosecond dynamical behavior is a method that may be used to systematically understand the factors that dictate protein stability. It had long been thought that the T_*g*_ of an excipient alone would dictate protein stability in a glass, but Cicerone et al.[115], [116] showed that this is not the case for trehalose–glycerol binary glasses. Dirama et al. [117] reported MD simulations of trehalose–glycerol binary glasses. They found that the formation of robust intermolecular hydrogen bonds between glycerol and trehalose are responsible for the suppression of dynamics in the glass.

These studies suggest that the dynamics of glassy matrices directly impacts the stability of the embedded proteins. The dynamics of glassy matrices can be classified in three different regimes depending on the time scales when they occur: *α*-relaxation, *β*_*slow*_-relaxation, and *β*_*fast*_-relaxation. The *α*-relaxation is the slowest relaxation and has been invoked in the ‘vitrification’ hypothesis of glassy systems [78], [118]. The high-frequency relaxations are the *β*-relaxations, where *β*_*slow*_-relaxation results in small amplitude motions that occur in the microsecond to millisecond time scales in sugar glasses. The *β*_*fast*_-relaxation occurs in the picosecond to nanosecond time scale and is associated with intra- and intermolecular collisions [81]. The *β*_*fast*_-relaxation regime can be accessed with neutron scattering, as presented in the work of Cicerone and Douglas [81], who showed that the  <*x*^2^ > in *β*-relaxation processes correlates well with the stability of proteins embedded in sugar–glass matrices. They showed that other dynamic relaxations and the replacement of water molecules by additives cannot account for the observed protein stability. A linear correlation between  <*x*^2^ > and aggregation or chemical degradation rates is observed for various freeze-dried proteins in sugar glasses spanning time scales that vary up to 15 orders of magnitude [81]. This result is presented in Fig. 9B, which shows that neutron scattering measurements can be a valuable tool to study the stability of proteins in amorphous phases. A fluorescence-based technique has been developed as a neutron surrogate of the  <*x*^2^ > measurements [119]. This technique is promising as a bench top approach to predict protein stability in amorphous phases. The current standing hypotheses regarding the mechanisms of stabilization of proteins in solid formulations are summarized in a review article [120].

**Fig. 10:**
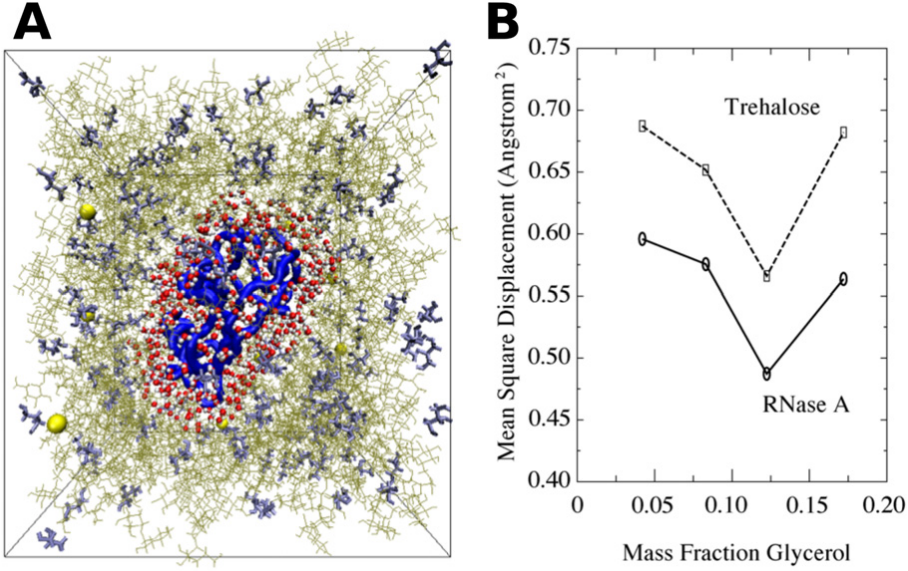
A. Snapshot of hydrated ribonuclease A in glycerol and trehalose glass. Protein density is ∼7 mM. B.  <*x*^2^ > from MD simulations for ribonuclease A and trehalose in a binary glass. A minimum  <*x*^2^ > is found at a particular mass fraction of glycerol, in agreement with experimental data [115] .

Source: Adapted with permission from Ref. [121] . Copyright 2006 American Chemical Society.

MD simulations have been used to investigate the dynamics of a hydrated proteins encased in glycerol, unary trehalose, and binary glycerol–trehalose glasses [121]. An example configuration derived from 10  ns MD trajectories of the protein ribonuclease A (RNase) in hydrated amorphous periodic boxes at 325 K is shown in Fig. 10A. A comparison of the  <*x*^2^ > of protein hydrogens to trehalose hydrogens as a function of glycerol content is shown in Fig. 10B. The dynamics of the protein follows the average dynamics of the trehalose molecules in the glass. The resulting  <*x*^2^ > has a minimum value at a particular mass fraction of glycerol, consistent to the experimental trend observed in dehydrated glycerol–trehalose protein-free glasses [115]. The mole fraction corresponding to maximum rigidity occurs at 12.2% of glycerol in the MD simulation, which has a much higher concentration of protein (7  mM, M = mol/L) than in the experimental measurements. This result is expected, because the protein content affects the amount of glycerol required to maximize the recovery of biological activity in binary glasses as shown experimentally [122].

**Fig. 11:**
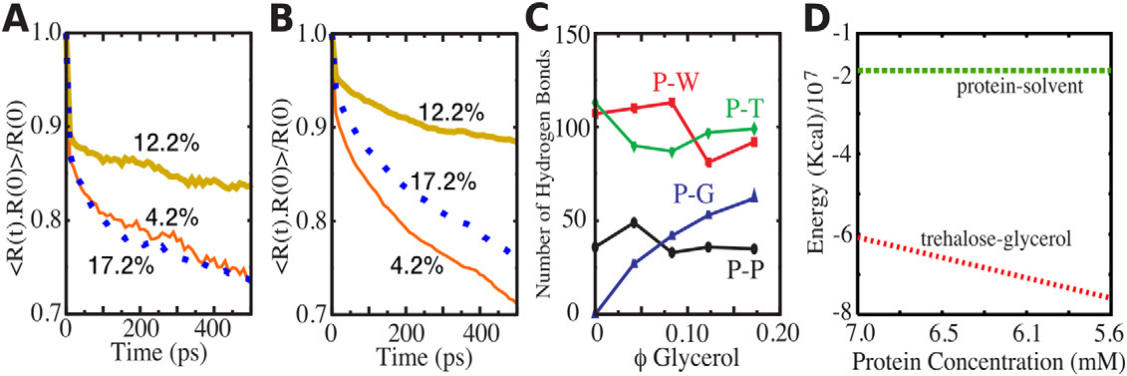
Origins of protein dynamics in glassy systems. Residence time-correlation functions for A glycerol and B water for a 4 Å water shell from the surface of the protein as a function of glycerol content. C. Average hydrogen bonds between protein and: protein (P-P), water (P-W), trehalose (P-T), and glycerol (P-G). D. Interaction energies for protein–solvent and trehalose–glycerol.

Source: Adapted with permission from reference [121] . Copyright 2006 American Chemical Society.

To characterize the molecular determinants that lead to the suppression of protein dynamics, the residence times of glycerol and water molecules about the protein surface are shown in Fig. 11A and B respectively. On average, glycerol and water molecules close to the protein surface stay in the vicinity longer in the 12.2% glycerol system compared to the other binary glycerol–trehalose glasses. As shown in Fig. 11C, analysis of the number of protein–solvent hydrogen bonds indicates that there may be a preference for protein–trehalose hydrogen bonds over protein–water hydrogen bonds in the 12.2% glycerol, where the protein and glass dynamics is maximally suppressed. This result is consistent with the idea that protein preservation is dictated by the exclusion of plasticizing water from the protein surface by trehalose.

The energy for a binary glycerol–trehalose glass as a function of protein concentration is presented in Fig. 11D. Values for trehalose–glycerol energies were estimated by extrapolation of the results obtained from the 7  mM simulation study using the energetics of a subvolume of trehalose–glycerol molecules removed from the protein surface (e.g., energetics per mole of pairwise trehalose–glycerol interactions multiplied by the mole of interactions in the subvolume). Clearly, the presence of a protein is a perturbation to the glassy matrix, but the protein–solvent energy is less than 10^6^ times than the glycerol–trehalose interaction energy. Thus, under the experimental conditions where maximal recovery of protein function occurs upon reconstitution from a binary glycerol–trehalose glass, the energetics of the glass far outweigh the energetics of the protein–solvent interface. The detailed microscopic picture that emerges is that protein dynamics are suppressed mainly by the inertia of the bulk glass and to a lesser extent by specific interactions at the protein–solvent interface.

The systematic study of the effect of cosolvents and proteins in non-aqueous environments is an active area of research with obvious technical applications in the biotechnology industry. Besides fundamental scientific discoveries, the overall goal of these experimental and theoretical studies is to understand the molecular mechanisms of protein stability in a manner that can aid the design of formulations to stabilize proteins. A key advance, summarized in this section, is the recognition that molecular motions on the picosecond time scale are relevant to the long-term stability of proteins. Clearly, the elucidation of the role of cosolvents on protein dynamics and stability constitutes a continuing experimental and theoretical challenge in which many scientifically interesting and industrially relevant insights await study. Finally, new facilities and instrumentation that directly focus on the specific needs to study dynamics of biological systems have been developed, which have the potential to make a dramatic impact on our knowledge of protein dynamics in the amorphous phase [123]

## 6 Conclusions and Outlook

In conclusion, neutron scattering is a powerful technique to study structure and dynamics of materials in various phases. Its uniqueness comes from the distinct interaction of the incident neutrons with the atomic nuclei and, in particular, with hydrogen. This feature of neutrons allows the use of contrast matching techniques for structural studies and neutron spectroscopy measurements for dynamic studies at energies that are relevant for biological systems. Neutron scattering is largely used for studies in solution, but its capabilities can be extended to the study of crowded proteins in solution, frozen conditions, and freeze-dried powders. Although these environments are of interest for the biopharmaceutical industry, they are not amenable to most other biophysical characterization techniques.

SANS has provided insights into the morphology of proteins in amorphous phases. By the use of SANS, it has been shown that proteins phase separate into an ice phase and a protein-concentrated phase when cooled below their freezing temperature; therefore, proteins arrange at distances much closer than in the liquid solution, regardless of the initial protein concentration. Additionally, SANS can be used to perform in situ studies on both the effects of additives, such as cryoprotectants, and the structure of proteins after freezing and thawing. No structural differences are observed between the lysozyme frozen solution and the freeze-dried lysozyme powder, but these results should be investigated in other relevant systems and conditions.

Besides using neutron scattering for morphology studies, neutron scattering can be used to probe the energies of the nuclei in the system and thus their motions. We briefly introduced the theoretical considerations for a neutron spectroscopy experiment and the role of molecular dynamics simulations to analyze and directly compare experimental neutron scattering data to computational models. In particular, neutron scattering can provide the mean-square displacement of hydrogens, which has been correlated to stability data of proteins and enzymes in amorphous phases under various conditions. Moreover, combining experimental neutron data with molecular dynamics simulations has provided deeper insights into the molecular interactions and energies of the different components. Thus, a better understanding of the microscopic mechanism of stabilization in amorphous glasses has emerged.

Although tremendous progress has been made in the field of stabilization of proteins in amorphous phases, there are still aspects of the field that can be further explored with neutron scattering. For example, the stability of the lyophilized product can be affected by the residual water content [124], [125], which varies for each protein and formulation studied. Neutron scattering experiments have been performed to evaluate the role of water content on the protein dynamics of lyophilized protein powders [83], [99], [126]. However, a generalized picture of how these neutron spectroscopy measurements correlate with long-term stability of freeze-dried proteins is needed. Therefore, it is imperative that neutron scattering measurements are accompanied by stability data that provides correlations and aids in the design of stable lyophilized products.

In addition, neutron scattering experiments should be accompanied by molecular simulations when possible to validate models representing the structure and dynamics of proteins. Although some limitations still exist on the computational models that can be handled by the available force fields and computational resources, further advancements in the field and the technology will allow simulations that provide a more in-depth understanding of the molecular basis that leads to stable proteins in solid forms.

Although sugars and polyols, such as sorbitol and glycerol, have shown to improve the storage stability of lyophilized powders, amino acids can also exert a positive effect on the stability of lyophilized powders [127]. Neutron scattering can provide insights on the stabilization mechanism of these excipients at the molecular level, not only by measuring their effect on glass dynamics, but also by evaluating their influence on structural arrangements of proteins.

Finally, the stresses that proteins undergo during the freezing and drying processes should be reproduced as close as possible to those conditions typically encountered in a lyophilization industrial setting. Neutron scattering has the advantage of being suitable for a wide range of instrumentation and conditions. Thus, closed cycle refrigerators, cryostats, and humidity chambers, among other equipment are available at most neutron facilities to produce different sample environments and stresses encountered during lyophilization.

Certain commercial equipment, instruments, materials, suppliers, or software are identified in this paper to foster understanding. Such identification does not imply recommendation or endorsement by the National Institute of Standards and Technology, nor does it imply that the materials or equipment identified are necessarily the best available for the purpose.

MMC acknowledges financial support from the NIST biomanufacturing initiative. This work benefitted from CCP-SAS software developed through a joint EPSRC (EP/K039121/1) and NSF (CHE-1265821) grant.

## Figures and Tables

**Table 1:** Neutron scattering cross sections for some biologically relevant atoms [12] .

Nucleus	Cross section (10^ −24^ cm^2^)		
	Coherent	Incoherent	Total
^1^H	1.7583	80.27	82.03
^2^H	5.592	2.05	7.64
^12^C	5.559	0	5.559
^14^N	11.03	0.5	11.53
^16^O	4.232	0	4.232
^32^S	0.988	0	0.988
^56^Fe	12.42	0	12.42
